# Personal

**Published:** 1910-08

**Authors:** 


### PERSONAL.

---

Dr. F. Park Lewis, of Buffalo, is the author of an article entitled "The Parable of the Unwise Farmer," published in *The Survey,* week of June 4, 1910. The purpose of the paper is to show the folly of the state in dealing with our defectives in public institutions, and especially in its present neglect of preventable diseases.

---

Dr. William A. Howe, of Phelps, has been appointed deputy commissioner of health of the State of New York. This is an office created by the last legislature and has been appropriately filled by the health commissioner in the appointment of Dr. Howe.

---

Dr. Herman E. Hayd, of Buffalo, with a party of friends, has been making a motor car tour to Spring Lake, N. J., which occupied a fortnight or more early in July. It is needless to add that fine sport was the result.

---

Dr. Uri C. Lynde, of Buffalo, who was seriously injured in leaving a street car on Niagara Street early in the summer, is reported to be well on the road toward recovery. The Journal extends its sympathy to Dr. Lynde and hopes his recovery will be complete very soon.

---

Dr. Orange G. Pfaff, of Indianapolis, on his way from New York to his home city in an automobile, accompanied by his wife and two sons, was the victim of an accident at Little Falls, June 24, 1910. Turning part way from the road to admit the passage of another car, Dr. Pfaff's automobile was overturned in the soft ground. He was pinned under the car, his left leg was broken, and the patella was fractured. He is at Saint Vincent's Hospital, Indianapolis, well on the way to recovery.

---

Dr. Robert E. Hoyt, passed Assistant Surgeon U. S. Navy, has been assigned to the Buffalo Naval Recruiting Station, relieving passed assistant surgeon Bert F. Jenness, who has been assigned to the naval hospital at Portsmouth, Va.

---

Dr. Charles N. Palmer, of Lockport, one of the best known physicians in Western New York, was seriously injured in a motor car accident at Cambria early in July. Pneumonia developed and for a time the result seemed doubtful but later advices indicate that he will recover. Dr. Palmer has the sympathy and congratulations of the Journal.

---

Dr. Stephen Yates Howell, of Buffalo, went to the seashore in July, where he spent the last half of the month.

---

Dr. and Mrs. Max C. Breuer, of Buffalo, made a motor car tour in the early summer, along the Atlantic Coast and through the White Mountains.

---

Dr. Charles Cary, of Buffalo, Professor of Clinical Medicine of the University, is touring a part of Europe in a motor car during the present summer.

---

Dr. Lucien Howe, of Buffalo, was granted the honorary degree of doctor of science by Bowdoin College at its annual commencement held in June, 1910.

---

Dr. Francis E. Fronczak, health commissioner of Buffalo, has been in Europe for a few weeks whither he went as the official delegate of the health department to the Congress of School Hygiene at Paris. He also inspected the open air schools of various countries, spending a pleasant but busy six weeks abroad.

---

Dr. William Gaertner, of Buffalo, was elected vice-president of the State German-American Alliance during its recent annual meeting at Albany. He also was made chairman of the legislative committee of the alliance.

---

Dr. John H. Grant, of Buffalo, Chairman of the Board of Censors of the Medical Society of the County of Erie, Assistant Commissioner of Agriculture of the State of New York spent a month in Albany, during the early summer.

---

Dr. G. A. Hitzel, of Buffalo, who has been in Berlin and other German university cities for the past three months or more, engaged in study and recreation, will return early in August and resume his professional work.

